# Impacts of poultry manure and biochar amendments on the nutrients in sweet potato leaves and the minerals in the storage roots

**DOI:** 10.1038/s41598-024-67486-9

**Published:** 2024-07-18

**Authors:** Taiwo Michael Agbede, Adefemi Oyewumi, Gabriel Kehinde Agbede, Aruna Olasekan Adekiya, Ojo Timothy Vincent Adebiyi, Thomas Adebayo Abisuwa, Justin Orimisan Ijigbade, Catherine Temitope Ogundipe, Adeola Oluwatoyin Wewe, Oluwabukola Dorcas Olawoye, Ehiokhilen Kevin Eifediyi

**Affiliations:** 1 Department of Agronomy, Adekunle Ajasin University, P.M.B. 001, Akungba-Akoko, Ondo State Nigeria; 2 Department of Agricultural Technology, Rufus Giwa Polytechnic, P.M.B. 1019, Owo, Ondo State Nigeria; 3 Bursary Department, Rufus Giwa Polytechnic, P.M.B. 1019, Owo, Ondo State Nigeria; 4 Agriculture Program, College of Agriculture, Engineering and Science, Bowen University, P.M.B. 284, Iwo, Osun State Nigeria; 5 Crop and Soil Sciences Programme, College of Agricultural Sciences, Landmark University, P.M.B. 1001, Omu-Aran, Kwara State Nigeria; 6 Department of Agricultural and Bio-Environmental Engineering Technology, Rufus Giwa Polytechnic, P.M.B. 1019, Owo, Ondo State Nigeria; 7 Department of Languages, Rufus Giwa Polytechnic, P.M.B. 1019, Owo, Ondo State Nigeria; 8 Department of Agronomy, University of Ilorin, P.M.B. 1515, Ilorin, Kwara State Nigeria

**Keywords:** Biological techniques, Environmental sciences, Health care, Physics

## Abstract

Poultry manure (PM) has demonstrated its potential to enhance crop nutritional quality. Nevertheless, there remains a dearth of knowledge regarding its synergistic effects when combined with wood biochar (B) on the nutrient concentrations in sweet potato leaves (*Ipomoea batatas* L.) and the mineral content stored in sweet potato storage roots. Hence, a two-year field trial was undertaken during the 2019 and 2020 cropping seasons in southwestern Nigeria, spanning two locations (Owo—site A and Obasooto—site B), to jointly apply poultry manure and wood biochar as soil amendments aimed at enhancing the nutritional quality of sweet potato crop. Each year, the experiment involved different combinations of poultry manure at rates of 0, 5.0, and 10.0 t ha^−1^ and biochar at rates of 0, 10.0, 20.0, and 30.0 t ha^−1^, organized in a 3 × 4 factorial layout. The results of the present study demonstrated that the individual application of poultry manure (PM), biochar (B), or their combination had a significant positive impact on the nutrient composition of sweet potato leaves and minerals stored in the sweet potato storage roots, with notable synergistic effects between poultry manure and biochar (PM × B) in enhancing these parameters. This highlights the potential of biochar to enhance the efficiency of poultry manure utilization and improve nutrient utilization from poultry manure. The highest application rate of poultry manure at 10.0 t ha^−1^ and biochar at 30.0 t ha^−1^ (PM10 + B30), resulted in the highest leaf nutrient concentrations and mineral composition compared to other treatments at both sites. Averaged over two years, the highest application rate of poultry manure at 10.0 t ha^−1^ and biochar at 30.0 t ha^−1^ (PM10 + B30) significantly increased sweet potato leaf nutrient concentrations: nitrogen by 88.2%, phosphorus by 416.7%, potassium by 123.8%, calcium by 927.3%, and magnesium by 333.3%, compared to those in the control (PM0 + B0). The same treatment increased the concentration of sweet potato root storage minerals: phosphorus by 152.5%, potassium by 77.4%, calcium by 205.5%, magnesium by 294.6%, iron by 268.4%, zinc by 228.6%, and sodium by 433.3%, compared to the control. The highest application rate of poultry manure at 10.0 t ha^−1^ and biochar at 30.0 t ha^−1^ yielded the highest economic profitability in terms of gross margin (44,034 US$ ha^−1^), net return (30,038 US$ ha^−1^) and return rate or value-to-cost ratio (VCR) (263). The results suggested that the application of poultry manure at 10 t ha^−1^ and biochar at 30 t ha^−1^ is economically profitable in the study areas and under similar agroecological zones and soil conditions.

## Introduction

Agriculture remains a fundamental pillar of global food security and the livelihoods of millions of people worldwide. In Nigeria, as in many other countries, the cultivation of crops is central to the economy and sustenance of the population. In this context, achieving optimal crop yields and nutritional quality while maintaining soil health is a persistent challenge, particularly in regions with degraded soils. Southwest Nigeria, characterized by a predominance of sandy soils, faces significant agricultural challenges due to soil infertility, nutrient depletion, and a rapidly growing population's increasing demand for food^1^. Sweet potato (*Ipomoea batatas* L.) is an important staple crop in the region, contributing significantly to food security and the economy^2^. As such, there is an urgent need to address the dwindling crop productivity in this region through sustainable agricultural practices.

The nutritional quality and yield of sweet potato crops are intricately linked to nutrient availability and uptake by the plant^3^. Poultry manure and biochar have emerged as potential soil amendments to enh

ance soil fertility, nutrient cycling, and crop productivity^4^. Poultry manure, a rich source of organic matter and essential nutrients, can improve soil structure and fertility^5^, while biochar, a carbon-rich material produced through pyrolysis of organic matter, can enhance nutrient retention, water-holding capacity, and microbial activity in the soil^6,7^. Both of these amendments have gained recognition for their potential to enhance crop performance and reduce environmental impacts in sustainable agriculture.

However, despite the growing interest in the use of poultry manure and biochar, there is a significant knowledge gap, particularly in Southwest Nigeria, regarding the effects of these amendments on leaf nutrient composition and storage root mineral reserves in sweet potato crops. The nutrient content of sweet potato leaves is not only essential for the crop's nutritional value but also an indicator of the plant's health and nutrient status^8^. Understanding how poultry manure and biochar influence these factors is pivotal for designing effective soil management strategies. This knowledge gap underscores the need for research investigating the impacts of poultry manure and biochar, both individually and in combination, on leaf nutrient composition and storage root mineral reserves in sweet potato crops in Southwest Nigeria. Addressing this gap will not only contribute to improved crop yields and nutritional quality but also promote sustainable agriculture and soil management practices in the region. The potential benefits of this research are twofold: first, it will provide valuable insights into how poultry manure and biochar can be harnessed to enhance crop nutritional quality, particularly in degraded sand soils and sandy loam soils; second, it will contribute to a growing body of knowledge that can be applied to similar agricultural contexts globally.

In this study, our primary hypothesis was that the incorporation of poultry manure and biochar into the soil would lead to substantial enhancements in the nutritional qualities of sweet potato. Additionally, we anticipated that these amendments would increase the concentrations of essential inorganic minerals crucial for human nutrition in the leaves and root storage of the sweet potato crop, compared to those in the untreated control. Hence, the objective of this research was to evaluate the impact of poultry manure and biochar application on the leaf nutrient composition and minerals stored in the storage roots of sweet potato cultivated in severely degraded tropical agricultural soils located in southwest Nigeria.

## Results

### Initial soil physical and chemical properties, climatic conditions of the sites, and chemical composition of poultry manure and biochar used in the experiment

**Figure 1:**
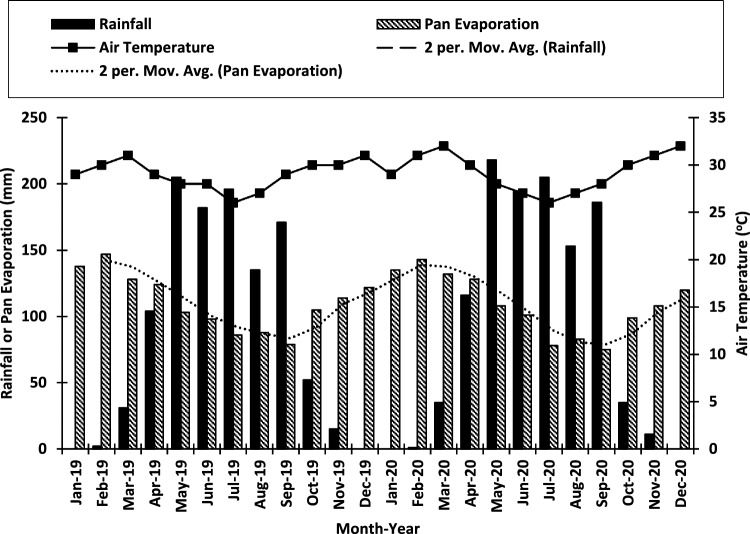
Meteorological data for Owo area 2019–2020. Total rainfall: 1093 mm in 2019; 1154 mm in 2020. Total evaporation: 1332 mm in 2019 and 1310 mm in 2020. Average air temperature: 29.0 °C in 2019 and 29.3 °C in 2020. Note: 2 per. Mov. Avg. means two periods moving average trendline (Agbede et al.^9^).

The initial soil physical and chemical properties (0–15 cm depth) at site A and site B prior to experimentation in 2019 indicated low levels of organic carbon, total nitrogen, available phosphorus, exchangeable potassium, calcium, and magnesium (Table 1). The annual rainfall totals for 2019 and 2020 were 1093 and 1154 mm, respectively. The total evaporation amounts were 1332 mm in 2019 and 1310 mm in 2020, while the average air temperature was 29.0 °C in 2019 and 29.3 °C in 2020 (Fig. 1). In terms of chemical characteristics, poultry manure was found to be slightly acidic and exhibited elevated levels of nitrogen (N), phosphorus (P), and various micronutrients in comparison to biochar. Conversely, biochar displayed slightly alkaline properties and had higher concentrations of organic carbon (OC), potassium (K), calcium (Ca), and magnesium (Mg), and C:N ratios than poultry manure (Table 2).

**Table 1:** Mean ± standard deviation of soil physical and chemical properties (0–15 cm depth) of the site A and site B prior to experimentation in 2019; Low: nutrient content value below the critical level recommended for crop production; High: nutrient content value above the critical level recommended for crop production. Three replicates were used for the analysis in the table.

Property	Site A	Class	Site B	Class
Sand (g kg^−1^)	920 ± 5.8		760 ± 4.3	
Silt (g kg^−1^	30 ± 0.1		130 ± 0.5	
Clay (g kg^−1^)	50 ± 0.2		110 ± 0.4	
Textural class	Sand		Sandy loam	
Bulk density (Mg m^-3^)	1.61 ± 0.04	High	1.58 ± 0.03	High
pH (water)	5.51 ± 0.2	Moderately acidic	5.52 ± 0.3	Moderately acidic
Organic carbon (g kg^−1^)	12.3 ± 0.02	Low	13.4 ± 0.02	Low
Total N (g kg^−1^)	1.2 ± 0.01	Low	1.4 ± 0.01	Low
Available P (mg kg^−1^)	6.75 ± 0.3	Low	8.12 ± 0.4	Low
Exchangeable K (cmol kg^−1^)	0.11 ± 0.01	Low	0.12 ± 0.01	Low
Exchangeable Ca (cmol kg^−1^)	1.35 ± 0.02	Low	1.51 ± 0.02	Low
Exchangeable Mg (cmol kg^−1^)	0.37 ± 0.01	Low	0.39 ± 0.01	Low
Exchangeable Na (cmol kg^−1^)	0.11 ± 0.01	Low	0.13 ± 0.01	Low
Cu (mg kg^−1^)	0.41 ± 0.01	Low	0.48 ± 0.01	Low
Fe (mg kg^−1^)	3.30 ± 0.03	–	3.50 ± 0.03	Low
Mn (mg kg^−1^)	2.97 ± 0.02	Low	3.42 ± 0.02	Low
Zn (mg kg^−1^)	0.31 ± 0.01	Low	0.38 ± 0.01	Low

**Table 2:** Chemical composition of poultry manure and biochar used in the experiment.

Property	Poultry manure		Biochar	
	2019	2020	2019	2020
Electrical conductivity (dS m^−1^)	0.98	0.97	3.84	3.86
pH (water)	6.25	6.28	7.86	7.89
Ash (%)	12.1	12.2	8.32	8.29
Organic C (%)	22.3	22.1	55.7	55.5
Nitrogen (%)	2.89	2.88	0.85	0.86
C/N	7.72	7.67	65.5	64.5
Phosphorous (%)	1.34	1.32	0.38	0.35
Potassium (%)	1.57	1.59	1.92	1.89
Calcium (%)	0.92	0.94	4.63	4.60
Magnesium (%)	0.46	0.47	3.78	3.75
Copper (mg kg^−1^)	3700	3710	130	131
Iron (mg kg^−1^)	112	114	104	105
Manganese (mg kg^−1^)	2100	2086	680	670
Zinc (mg kg^−1^)	2400	2380	80	78
Sodium (mg kg^−1^)	2700	2710	2100	2100

### Impact of year, site, poultry manure and biochar and their combination on soil chemical properties

The effects of site, poultry manure and biochar on soil chemical properties in 2019 and 2020 are shown in Table 3, and Supplementary Material Figure S1 and Figure S2. In both years (2019 and 2020), site, poultry manure and biochar played a significant role in influencing the soil chemical properties when studied as individual factors. In both years (2019 and 2020), poultry manure and biochar applied alone significantly increased the soil pH, organic carbon (OC), total nitrogen (TN) (Table 3, Supplementary Material Figure S1), available phosphorus (P), exchangeable (K), exchangeable (Ca) and exchangeable (Mg) (Table 3, Supplementary Material Figure S2), and the concentration increased with increasing poultry manure and biochar application rates. Poultry manure increased the soil pH, OC, TN (Table 3, Supplementary Material Figure S1), P, K, Ca and Mg (Table 3, Supplementary Material Figure S2) at rate ranging from 0 to 10.0 t ha^−1^. Similarly, biochar increased the soil pH, OC, TN (Table 3, Supplementary Material Figure S1), P, K, Ca and Mg (Table 3, Supplementary Material Figure S2) at rate ranging from 0 to 30.0 t ha^−1^. The application rate of poultry manure at 10.0 t ha^−1^ + biochar at 30.0 t ha^−1^ (PM10 + B30) had the greatest effect on the soil chemical properties among all the treatments. The control (no application of poultry manure or biochar) had the lowest values of soil chemical properties. The second year (2020) had higher concentrations of soil pH, OC, TN, P, K, Ca and Mg than the first year (2019) (Table 3).

**Table 3:** Impact of year, site, poultry manure and biochar, and their combination on soil pH, organic carbon (OC), total nitrogen (TN), phosphorus (P), potassium (K), calcium (Ca) and magnesium (Mg).

Year/site	Poultry manure (t ha^−1^)	Biochar (t ha^−1^)	pH (water)	OC (%)	Total N (%)	Available P (mg kg^−1^)	Exchangeable K (cmol kg^−1^)	Exchangeable Ca (cmol kg^−1^)	Exchangeable Mg (cmol kg^−1^)
2019									
A	0.0	0.0	5.51	1.14	0.11	6.5	0.09	1.21	0.34
	0.0	10.0	6.24	1.75	0.13	7.2	0.14	1.66	0.71
	0.0	20.0	6.54	1.87	0.17	8.4	0.18	2.11	0.79
	0.0	30.0	6.78	2.24	0.19	9.6	0.30	2.56	1.01
	5.0	0.0	5.96	1.32	0.13	8.6	0.11	1.36	0.54
	5.0	10.0	6.40	1.81	0.17	12.8	0.16	1.82	0.65
	5.0	20.0	6.65	2.11	0.21	17.5	0.23	2.27	0.89
	5.0	30.0	6.89	2.57	0.25	21.4	0.34	2.72	1.12
	10.0	0.0	6.18	1.54	0.15	10.8	0.13	1.51	0.62
	10.0	10.0	6.51	1.93	0.19	14.9	0.20	1.98	0.81
	10.0	20.0	6.70	2.29	0.23	19.8	0.27	2.43	0.97
	10.0	30.0	6.97	2.05	0.27	24.1	0.38	2.88	1.21
B	0.0	0.0	5.52	1.25	0.13	7.2	0.10	2.21	0.36
	0.0	10.0	6.29	1.72	0.15	7.9	0.15	3.06	0.83
	0.0	20.0	6.59	2.21	0.19	8.6	0.19	3.92	0.91
	0.0	30.0	6.83	2.72	0.20	9.3	0.31	4.79	1.13
	5.0	0.0	6.01	1.43	0.15	9.7	0.12	2.49	0.66
	5.0	10.0	6.45	1.90	0.20	14.8	0.17	3.35	0.72
	5.0	20.0	6.59	2.21	0.19	8.6	0.19	3.92	0.91
	5.0	30.0	6.94	2.91	0.29	25.5	0.35	5.08	1.25
	10.0	0.0	6.23	1.61	0.17	12.3	0.14	2.78	0.74
	10.0	10.0	6.56	2.08	0.22	17.6	0.21	3.64	0.93
	10.0	20.0	6.75	2.57	0.27	22.9	0.28	4.50	1.09
	10.0	30.0	7.02	3.08	0.31	28.2	0.39	5.39	1.37
2020									
A	0.0	0.0	5.49	1.02	0.10	5.3	0.08	1.16	0.32
	0.0	10.0	6.47	1.86	0.14	8.4	0.16	1.71	0.76
	0.0	20.0	6.77	1.98	0.18	9.6	0.20	2.16	0.84
	0.0	30.0	7.01	2.35	0.20	10.3	0.32	2.61	1.07
	5.0	0.0	6.18	1.43	0.15	10.9	0.12	1.41	0.59
	5.0	10.0	6.63	1.93	0.19	15.5	0.18	1.88	0.71
	5.0	20.0	6.86	2.25	0.23	20.5	0.24	2.34	0.93
	5.0	30.0	7.12	2.83	0.27	25.4	0.36	2.78	1.19
	10.0	0.0	6.47	1.86	0.14	8.4	0.16	1.71	0.76
	10.0	10.0	6.74	2.11	0.21	17.9	0.22	2.05	0.88
	10.0	20.0	6.93	2.44	0.25	22.8	0.28	2.50	1.01
	10.0	30.0	7.20	3.12	0.29	27.7	0.41	2.95	1.13
B	0.0	0.0	5.51	1.14	0.12	7.2	0.09	2.08	0.37
	0.0	10.0	6.52	1.99	0.16	6.9	0.17	3.10	0.86
	0.0	20.0	6.91	2.74	0.27	21.1	0.26	4.26	1.04
	0.0	30.0	7.06	3.11	0.21	9.7	0.33	4.83	1.16
	5.0	0.0	6.23	1.62	0.17	9.7	0.13	2.54	0.69
	5.0	10.0	6.68	2.18	0.22	16.1	0.19	3.40	0.75
	5.0	20.0	6.91	2.74	0.27	21.1	0.26	4.26	1.04
	5.0	30.0	7.17	3.34	0.31	26.4	0.37	5.14	1.28
	10.0	0.0	6.46	1.81	0.19	12.3	0.16	2.83	0.77
	10.0	10.0	6.79	2.37	0.24	18.8	0.23	3.69	0.96
	10.0	20.0	6.98	2.95	0.29	24.0	0.30	4.55	1.11
	10.0	30.0	7.25	3.59	0.33	29.6	0.42	5.45	1.40
	SE±		0.065	0.088	0.009	1.023	0.014	0.172	0.038
Year (Y)		*	*	*	*	*	*	*	*
Site (S)		*	*	*	*	*	*	*	*
PM		*	*	*	*	*	*	*	*
B		*	*	*	*	*	*	*	*
Y × S		*	*	*	*	*	*	*	*
Y × PM		*	*	*	*	*	*	*	*
Y × B		*	*	*	*	*	*	*	*
PM × B		*	*	*	*	*	*	*	*
PM × S		*	*	*	*	*	*	*	*
B × S		*	*	*	*	*	*	*	*
Y × S × PM × B		*	*	*	*	ns	*	*	*

*Significant difference at *P* < 0.05.

When examined as individual factors, year (Y) and site (S) exerted noticeable influences on the concentrations of soil pH, OC, N, P, K, Ca and Mg (Table 3). When studied as an individual factor, poultry manure significantly (*p* = 0.05) increased the concentrations of soil pH, OC, N, P, K, Ca and Mg. Similarly, the application of biochar as an individual factor, significantly (*p* = 0.05) increased the soil chemical properties (Table 3). Furthermore, the combined effects of various interactions-year and site (Y × S), year and poultry manure (Y × PM), year and biochar (Y × B), poultry manure and site (PM × S), biochar and site (B × S), and poultry manure and biochar (PM × B)-exhibited significant influences on soil pH, OC, N, P, K, Ca and Mg. When all four factors-year, site, poultry manure and biochar (Y × S × PM × B) were considered together, the interactions were significant for soil pH, OC, N, K, Ca and Mg, but the interactions were not significant for soil P (Table 3).

### Impact of year, site, poultry manure and biochar and their combination on the nutrient content of sweet potato leaves

Table 4, and Supplementary Material Figure S3 and Figure S4 illustrate the impacts of year, site, poultry manure and biochar on the nutrient concentrations in sweet potato leaves in 2019 and 2020. In both years, the interplay of site conditions, poultry manure, and biochar had significant effects on the nutrient concentrations in sweet potato leaves. Specifically, the application of either poultry manure or biochar, independently, led to a notable increase in the concentrations of N and P (Table 4, Supplementary Material Figure S3) as well as K, Ca, and Mg (Table 4, Supplementary Material Figure S4). Moreover, the concentration increased in a direct correlation with the quantity of poultry manure and biochar applied. In both 2019 and 2020, the nutrient concentrations in sweet potato leaves exhibited elevated levels of N and P (Table 4, Supplementary Material Figure S3) as well as K, Ca, and Mg (Table 4, Supplementary Material Figure S4) when poultry manure was applied at rates ranging from 0 to 10.0 t ha^−1^. Similarly, the application of biochar, within the range of 0 to 30.0 t ha^−1^, led to increased concentrations of N and P (Table 4, Supplementary Material Figure S3) as well as K, Ca, and Mg (Table 4, Supplementary Material Figure S4) in sweet potato leaves. Compared with those in the control group (no poultry manure or biochar application), the use of poultry manure, biochar in isolation, or their combined application at varying rates, resulted in significant increases in the levels of N, P, K, Ca, and Mg in sweet potato leaves, during both years. Notably, the coapplication of poultry manure and biochar at different levels yielded the highest leaf concentrations of N and P (Table 4, Supplementary Material Figure S3) as well as K, Ca, and Mg (Table 4, Supplementary Material Figure S4) in sweet potato, compared to the application of poultry manure or biochar individually. The peak concentrations of N, P, K, Ca, and Mg in sweet potato leaves were achieved when the highest application rates of both amendments were used (poultry manure at 10.0 t ha^−1^ in combination with biochar at 30.0 t ha^−1^) in both years, denoted as (PM10 + B30).

**Table 4:** Impact of year, site, poultry manure and biochar, and their combination on leaf nitrogen (N), phosphorus (P), potassium (K), calcium (Ca) and magnesium (Mg) concentrations in sweet potato.

Year/site	Poultry manure (t ha^−1^)	Biochar (t ha^−1^)	*N* (%)	*P* (%)	*K* (%)	*Ca* (%)	*Mg* (%)
2019							
A	0.0	0.0	2.15	0.06	1.45	0.10	0.05
	0.0	10.0	2.29	0.07	1.91	0.19	0.10
	0.0	20.0	2.31	0.08	2.35	0.46	0.13
	0.0	30.0	2.67	0.10	2.52	0.93	0.15
	5.0	0.0	2.32	0.08	1.68	0.13	0.07
	5.0	10.0	2.54	0.14	2.17	0.43	0.12
	5.0	20.0	3.12	0.20	2.61	0.69	0.16
	5.0	30.0	3.44	0.26	2.93	0.98	0.17
	10.0	0.0	2.61	0.10	1.98	0.31	0.09
	10.0	10.0	2.83	0.17	2.48	0.66	0.14
	10.0	20.0	3.41	0.23	2.83	0.80	0.18
	10.0	30.0	3.99	0.29	3.16	1.06	0.19
B	0.0	0.0	2.39	0.05	1.57	0.13	0.07
	0.0	10.0	2.40	0.08	2.04	0.26	0.17
	0.0	20.0	2.52	0.09	2.48	0.51	0.20
	0.0	30.0	2.88	0.11	2.65	0.99	0.22
	5.0	0.0	2.53	0.09	1.81	0.19	0.14
	5.0	10.0	2.75	0.15	2.30	0.50	0.19
	5.0	20.0	3.33	0.21	2.74	0.76	0.23
	5.0	30.0	3.65	0.27	3.06	1.05	0.24
	10.0	0.0	2.82	0.11	2.11	0.37	0.16
	10.0	10.0	3.04	0.18	2.61	0.72	0.21
	10.0	20.0	3.62	0.24	2.96	0.88	0.25
	10.0	30.0	4.20	0.30	3.29	1.14	0.26
2020							
A	0.0	0.0	2.05	0.06	1.36	0.08	0.04
	0.0	10.0	2.24	0.09	2.03	0.26	0.15
	0.0	20.0	2.46	0.10	2.47	0.53	0.18
	0.0	30.0	2.82	0.12	2.64	1.00	0.20
	5.0	0.0	2.47	0.10	1.80	0.20	0.12
	5.0	10.0	2.69	0.16	2.29	0.50	0.17
	5.0	20.0	3.27	0.22	2.73	0.76	0.21
	5.0	30.0	3.59	0.28	3.05	1.05	0.22
	10.0	0.0	2.76	0.12	2.11	0.38	0.14
	10.0	10.0	2.98	0.19	2.60	0.73	0.19
	10.0	20.0	3.56	0.25	2.95	0.87	0.23
	10.0	30.0	4.14	0.31	3.28	1.13	0.24
B	0.0	0.0	2.24	0.08	1.49	0.11	0.06
	0.0	10.0	2.92	0.15	2.24	0.46	0.22
	0.0	20.0	2.62	0.13	2.61	0.61	0.26
	0.0	30.0	2.98	0.15	2.78	1.08	0.28
	5.0	0.0	2.63	0.12	1.94	0.28	0.20
	5.0	10.0	2.86	0.21	2.43	0.58	0.25
	5.0	20.0	2.62	0.13	2.61	0.61	0.26
	5.0	30.0	3.76	0.31	3.19	1.13	0.30
	10.0	0.0	2.92	0.15	2.24	0.46	0.22
	10.0	10.0	3.17	0.25	2.74	0.81	0.27
	10.0	20.0	3.74	0.29	3.09	0.95	0.31
	10.0	30.0	4.32	0.35	3.41	1.20	0.33
	SE ±		0.084	0.012	0.075	0.049	0.010
Year (Y)			*	*	*	*	*
Site (S)			*	*	*	*	*
Poultry manure (PM)			*	*	*	*	*
Biochar (B)			*	*	*	*	*
Y × S			*	*	*	*	*
Y × PM			*	*	*	*	*
Y × B			*	*	*	*	*
PM × B			*	*	*	*	*
PM × S			*	*	*	*	*
B × S			*	*	*	*	*
Y × S × PM × B			*	*	*	*	*

*Significant difference at *P* < 0.05.

When studied as individual factors, both year (Y) and site (S) had a significant influence (*P* < 0.05) on the levels of leaf N, P, K, Ca and Mg in sweet potato plants (Table 4). Similarly, when examined as an individual factor, the application of poultry manure (PM) significantly (*P* < 0.05) increased the concentrations of leaf N, P, K, Ca, and Mg. Similarly, the application of biochar (B) as an individual factor, significantly (*P* < 0.05) increased the leaf nutrient concentrations of sweet potato plants (Table 4). Furthermore, the interactive effects of factors such as year and site (Y × S), year and poultry manure (Y × PM), year and biochar (Y × B), poultry manure and site (PM × S), biochar and site (B × S), and poultry manure and biochar (PM × B)-had significance impacts on leaf N, P, K, Ca, and Mg concentrations in sweet potato plants. Notably when all four factors-year, site, poultry manure and biochar (Y × S × PM × B) were considered together, their interactions remained significant (Table 4).

### Impact of year, site, poultry manure and biochar and their combination on the minerals accumulated in sweet potato storage roots

In both 2019 and 2020, site and the application of poultry manure and biochar had a significant impacts on the mineral content of sweet potato storage roots. In general, whether applied individually or in various combinations, poultry manure, biochar, or their mixtures resulted in a significant (*P* < 0.05) increase in the concentrations of minerals such as P (Table 5, Supplementary Material Figure S5), K, Ca, Mg (Table 5, Supplementary Material Figure S6), Fe, Zn, and Na (Table 5, Supplementary Material Figure S7) in sweet potato storage roots, compared to those in the control group, which received no poultry manure or biochar in both years. In both years, the application of poultry manure led to a significant (*P* < 0.05) increase in the concentrations of P (Table 5, Supplementary Material Figure S5), K, Ca, Mg (Table 5, Supplementary Material Figure S6), Fe, Zn, and Na (Table 5, Supplementary Material Figure S7) in sweet potato storage roots, with application rates ranging from 0 to 10.0 t ha^−1^. Similarly, biochar application significantly (*P* < 0.05) increased the concentrations of P (Table 5, Supplementary Material Figure S5), K, Ca, Mg (Table 5, Supplementary Material Figure S6), Fe, Zn, and Na (Table 5, Supplementary Material Figure S7) in sweet potato storage roots, with application rates ranging from 0 to 30.0 t ha^−1^. When poultry manure and biochar were combined at various levels, even higher concentrations of P (Table 5, Supplementary Material Figure S5), K, Ca, Mg (Table 5, Supplementary Material Figure S6), Fe, Zn, and Na (Table 5, Supplementary Material Figure S7) were detected in sweet potato storage roots than when poultry manure or biochar was applied alone. The highest mineral values for P, K, Ca, Mg, Fe, Zn, and Na were observed when poultry manure was applied at 10.0 t ha^−1^ + biochar at 30 t ha^−1^ (PM10 + B30), surpassing the other treatments. The control group displayed the lowest mineral nutrient levels in sweet potato storage roots in both years.

**Table 5:** Impact of year, site, poultry manure and biochar, and their combination on mineral phosphorus (P), potassium (K), calcium (Ca), magnesium (Mg), iron (Fe), zinc (Zn) and sodium (Na) of sweet potato tuber.

Year/site	Poultry manure (t ha^−1^)	Biochar (t ha^−1^)	P (mg 100 g^−1^)	K (mg 100 g^−1^)	Ca (mg 100 g^−1^)	Mg (mg 100 g^−1^)	Fe (mg 100 g^−1^)	Zn (mg 100 g^−1^)	Na (mg 100 g^−1^)
2019									
A	0.0	0.0	18.3	180.4	20.4	9.2	0.18	0.13	0.05
	0.0	10.0	20.7	205.9	24.2	11.2	0.23	0.15	0.06
	0.0	20.0	22.4	216.3	33.4	12.8	0.26	0.17	0.09
	0.0	30.0	24.9	228.4	47.3	14.6	0.30	0.20	0.10
	5.0	0.0	21.6	200.6	24.6	11.4	0.20	0.17	0.07
	5.0	10.0	27.5	221.7	34.5	16.5	0.28	0.22	0.12
	5.0	20.0	34.6	248.6	44.6	23.6	0.48	0.29	0.19
	5.0	30.0	38.7	293.0	61.4	32.9	0.63	0.36	0.26
	10.0	0.0	24.2	213.3	26.8	14.5	0.27	0.19	0.09
	10.0	10.0	31.2	236.8	39.8	21.7	0.40	0.25	0.15
	10.0	20.0	37.8	269.5	51.1	29.5	0.55	0.33	0.23
	10.0	30.0	41.6	305.6	62.7	34.5	0.67	0.41	0.29
B	0.0	0.0	19.9	187.8	25.3	10.3	0.20	0.15	0.08
	0.0	10.0	23.0	213.3	29.0	12.0	0.25	0.19	0.09
	0.0	20.0	25.3	223.7	38.1	13.9	0.29	0.21	0.12
	0.0	30.0	27.6	235.8	52.7	15.3	0.33	0.24	0.14
	5.0	0.0	23.4	208.0	29.4	12.6	0.23	0.22	0.11
	5.0	10.0	30.4	229.1	39.4	17.7	0.31	0.27	0.16
	5.0	20.0	37.8	256.0	49.2	24.4	0.51	0.35	0.23
	5.0	30.0	44.3	300.4	66.1	33.6	0.66	0.42	0.30
	10.0	0.0	26.5	220.7	31.5	15.6	0.30	0.25	0.13
	10.0	10.0	34.5	244.2	44.7	22.9	0.43	0.30	0.19
	10.0	20.0	41.2	276.9	55.9	30.8	0.58	0.39	0.27
	10.0	30.0	47.8	313.2	67.3	35.9	0.70	0.46	0.33
2020									
A	0.0	0.0	16.8	168.8	18.4	8.0	0.17	0.12	0.05
	0.0	10.0	22.9	223.3	27.7	13.5	0.25	0.17	0.07
	0.0	20.0	24.7	233.7	36.9	15.0	0.28	0.19	0.10
	0.0	30.0	27.4	245.8	50.8	17.2	0.32	0.22	0.12
	5.0	0.0	23.9	218.0	28.1	13.8	0.22	0.20	0.09
	5.0	10.0	29.8	239.1	38.0	18.9	0.30	0.25	0.14
	5.0	20.0	36.9	266.1	48.1	25.8	0.51	0.33	0.21
	5.0	30.0	41.5	310.4	64.9	35.4	0.65	0.40	0.28
	10.0	0.0	26.5	230.7	30.3	16.7	0.29	0.23	0.11
	10.0	10.0	33.5	254.2	43.3	23.8	0.42	0.28	0.17
	10.0	20.0	40.3	286.9	54.6	31.6	0.58	0.37	0.25
	10.0	30.0	44.8	323.0	66.2	36.9	0.69	0.44	0.31
B	0.0	0.0	18.1	175.9	23.5	9.1	0.19	0.14	0.07
	0.0	10.0	25.1	223.3	33.4	13.9	0.30	0.21	0.11
	0.0	20.0	28.1	233.7	42.3	15.8	0.34	0.24	0.14
	0.0	30.0	29.9	245.8	56.9	17.0	0.38	0.27	0.16
	5.0	0.0	26.2	218.0	33.6	14.5	0.28	0.25	0.15
	5.0	10.0	33.4	239.1	43.6	19.6	0.36	0.30	0.18
	5.0	20.0	40.6	266.1	53.4	26.3	0.56	0.39	0.25
	5.0	30.0	47.1	310.4	70.2	35.5	0.71	0.46	0.32
	10.0	0.0	29.3	230.7	35.7	17.5	0.35	0.28	0.15
	10.0	10.0	37.5	254.2	48.9	24.6	0.48	0.34	0.21
	10.0	20.0	44.0	286.9	60.1	32.6	0.63	0.43	0.29
	10.0	30.0	50.6	323.0	71.5	37.8	0.75	0.51	0.35
	SE ±		1.28	5.64	2.11	1.28	0.025	0.015	0.012
Year (Y)			*	*	*	*	*	*	*
Site (S)			*	*	*	*	*	*	*
Poultry manure (PM)			*	*	*	*	*	*	*
Biochar (B)			*	*	*	*	*	*	*
Y × S			*	*	*	*	*	*	*
Y × PM			*	*	*	*	*	*	*
Y × B			*	*	*	*	*	*	*
PM × B			*	*	*	*	*	*	*
PM × S			*	*	*	*	*	*	*
B × S			*	*	*	*	*	*	*
Y × S × PM × B			*	*	*	*	*	*	*

*Significant difference at *P* = 0.05.

When examined as an individual factor, year (Y) and site (S) exerted noticeable influences on the concentrations of P, K, Ca, Mg, Fe, Zn, and Na in the storage roots of sweet potato plants (Table 5). When studied as individual factors, the application of poultry manure (PM) and biochar (B) also affected mineral nutrition in sweet potato storage roots. Furthermore, the combined effects of various interactions-year and site (Y × S), year and poultry manure (Y × PM), year and biochar (Y × B), poultry manure and site (PM × S), biochar and site (B × S), and poultry manure and biochar (PM × B)-exhibited significant influences on all mineral nutrients in the sweet potato storage roots (Table 5). When all four factors-year, site, poultry manure and biochar (Y × S × PM × B) were considered together, their interactions remained significant (Table 5).

### Optimizing profitability. Impact of poultry manure and biochar treatments on sweet potato production

The total yields for the two years for the PM0 + B0, PM0 + B10, PM0 + B20, PM0 + B30, PM5 + B0, PM5 + B10, PM5 + B20, PM5 + B30, PM10 + B0, PM10 + B10, PM10 + B20, and PM10 + B30 treatments were: 11.2, 12.1, 14.5, 17.2, 16.0, 21.1, 26.2, 32.8, 18.9, 24.1, 29.3 and 35.8 t ha^−1^, respectively (Table 6). The cost of transportation for each level of poultry manure and biochar treatment (Table 6), increased with the rate of amendment. The use of poultry manure at 10 t ha^−1^ + biochar at 30 t ha^−1^ had the greatest gross return (US$44,034·ha^−1^) and net return (US$30,038 ha^−1^). This treatment was followed by poultry manure at 5 t ha^−1^ + biochar at 30 t ha^−1^ treatment with a gross return of US$40,344 ha^−1^ and a net return of US$26,375 ha^−1^. The lowest gross return (US$13,776 ha^−1^) was from the control (N is the Naira, Nigerian currency, 1US$ = 360.00N in year 1 (2019) and 1US$ = 370.00N in year 2 (2020). All levels of poultry manure and biochar and their combinations at various rates produced greater net profits than did the control. Compared with all other treatments, poultry manure at 10 t ha^−1^ + biochar at 30 t ha^−1^ was the most cost effective and profitable for sweet potato production, as evidenced by its high return rate or value-to-cost ratio of 263.

**Table 6:** Economics of producing sweet potato under each level of poultry manure and biochar tested in 2019 and 2020.

Treatment	Yield (t ha^−1^)	Gross return (US$ ha^−1^)	Production increase value (US$ ha^−1^)	Production increase (%)	Cost of transportation of poultry manure/biochar (US$ ha^−1^)	Net return over each fertilization (US$ ha^−1^)	Return rate or value/cost ratio of each fertilization
0 t ha^−1^ PM + 0 t ha^−1^ B	11.2	13,776	–	–	–	–	–
0 t ha^−1^ PM + 10 t ha^−1^ B	12.1	14,883	1107	8	52	1099	21
0 t ha^−1^ PM + 20 t ha^−1^ B	14.5	17,835	4059	29	75	3984	54
0 t ha^−1^ PM + 30 t ha^−1^ B	17.2	21,156	7380	54	93	7267	79
5 t ha^−1^ PM + 0 t ha^−1^ B	16.0	19,680	5904	43	45	5861	136
5 t ha^−1^ PM + 10 t ha^−1^ B	21.1	25,953	12,177	88	68	12,089	179
5 t ha^−1^ PM + 20 t ha^−1^ B	26.2	32,226	18,450	134	83	18,316	222
5 t ha^−1^ PM + 30 t ha^−1^ B	32.8	40,344	26,568	193	110	26,375	242
10 t ha^−1^ PM + 0 t ha^−1^ B	18.9	23,247	9471	69	54	9402	175
10 t ha^−1^ PM + 10 t ha^−1^ B	24.1	29,643	15,867	115	77	15,752	206
10 t ha^−1^ PM + 20 t ha^−1^ B	29.3	36,039	22,263	162	96	22,101	232
10 t ha^−1^ PM + 30 t ha^−1^ B	35.8	44,034	30,258	220	115	30,038	263

In the year of 2019, the price of fresh storage root yield of sweet potato was US$ 1.23 kg^−1^. In the year of 2020, the price of fresh storage root yield of sweet potato was US$ 1.23 kg^−1^. US$1 = N360 in 2019. US$1 = N370 in 2020.

## Discussion

The low levels of organic carbon, total nitrogen, available phosphorus, exchangeable potassium, calcium, and magnesium, high bulk density and low pH observed at both site A and site B prior to experimentation in 2019 (Table 1), could be attributed to soil degradation due to continuous cropping practices, suggesting significant soil fertility challenges in the region. The slightly acidic nature of poultry manure aligns with previous studies on the chemical composition of manure from various sources^10,11^. This acidity may influence soil pH when used as a fertilizer, affecting nutrient availability and microbial activity^12^. Additionally, the elevated levels of nitrogen and phosphorus in poultry manure are consistent with the nutrient-rich nature of animal-derived organic materials, suggesting its potential as an effective fertilizer for promoting plant growth^13^. In contrast, the alkaline properties of biochar have been associated with its potential to improve soil pH, soil structure, nutrient retention and cation exchange capacity^14^. The higher concentrations of organic carbon and essential nutrients such as potassium, calcium, and magnesium in biochar are indicative of its ability to enhance soil fertility and promote plant development^15,16^. The observed difference in the carbon-to-nitrogen ratio between poultry manure and biochar is noteworthy, because it influences decomposition dynamics and nutrient release in the soil^17^. A higher C:N ratio in biochar suggests slower decomposition rates, potentially leading to prolonged nutrient availability in the soil compared to poultry manure^18^. The higher C:N ratio of biochar suggests its potential to act as a stable, long-lasting carbon source in the soil, aiding in carbon sequestration and enhancing soil organic matter content^16^. The contrasting chemical characteristics of poultry manure and biochar highlight their distinct roles and potential benefits in agricultural practices. The strategic integration of these organic amendments based on their unique properties can contribute to sustainable soil management and crop productivity.

The second year (2020) had higher concentrations of soil pH, OC, TN, P, K, Ca and Mg than the first year (2019) due to the cumulative and residual effects of repeated applications of poultry manure and biochar. These amendments not only improved soil fertility in the first year but also continued to decompose and release nutrients over time, leading to enhanced nutrient retention and availability. Additionally, biochar has a persistent nature, which means its benefits compound over time, further improving soil properties in the subsequent year. The organic matter from the poultry manure decomposed and further enriched the soil, leading to greater nutrient concentrations in the second year.

The findings presented in this study shed light on the intricate dynamics of nutrient and mineral accumulation in sweet potato plants, particularly in their leaves and storage roots, under the influence of various factors such as year, site conditions, and the application of poultry manure and biochar. The comprehensive analysis of these factors underscores their individual and interactive roles in shaping the nutritional profile of sweet potato plants. The results demonstrated that both the year and site significantly impacted the nutrient concentrations in sweet potato leaves and storage roots due to the complex interaction of environmental, soil and climatic factors that vary by location and over time. This underscores the importance of considering site-specific and temporal factors in soil fertility management. These findings are consistent with previous studies highlighting the sensitivity of crop nutrient uptake to environmental variations and site-specific factors^19,20^.

The findings presented in this study demonstrated a significant impact of poultry manure and biochar on the nutrient content of sweet potato leaves during 2019 and 2020. The application of poultry manure and biochar, either individually or in combination, led to notable increases in the concentrations of N, P, K, Ca, and Mg in sweet potato leaves. This finding aligns with existing research indicating that organic amendments, such as poultry manure and biochar, can enhance nutrient concentrations in plant tissues^21,22^. Averaged over two years, the application of the highest rate of poultry manure at 10.0 t ha^−1^ and biochar at 30.0 t ha^−1^ (PM10 + B30) significantly increased sweet potato leaf nutrient concentrations: nitrogen by 88.2%, phosphorus by 416.7%, potassium by 123.8%, calcium by 927.3%, and magnesium by 333.3%; compared to those in the control (PM10 + B30). The observed increase in N, P, K, Ca, and Mg levels in sweet potato leaves corresponds with previous studies highlighting the positive effects of poultry manure and biochar on nutrient availability in soils^23,24^. Gune et al^25^ reported that the synergistic effect of application of poultry manure and biochar resulted in the highest N, P and K concentrations in lettuce plants, which is consistent with the concept of nutrient complementarity between organic amendments. This underscores the potential of integrated nutrient management strategies for sustainable agriculture, emphasizing the importance of combining poultry manure and biochar to maximize nutrient benefits in nutrient-deficient soils^26^.

Furthermore, the mineral content in sweet potato storage roots was significantly influenced by year, site conditions, and the application of poultry manure and biochar. The results indicated that the addition of poultry manure and biochar, either alone or in combination, resulted in increased concentrations of essential minerals such as phosphorus, potassium, calcium, magnesium, iron, zinc, and sodium in sweet potato storage roots. The highest mineral values were achieved when poultry manure and biochar were combined at optimal rates, suggesting the potential for integrated nutrient management strategies to maximize nutritional quality. Pooled over two years, the application of the highest rate of poultry manure at 10.0 t ha^−1^ and biochar at 30.0 t ha^−1^ (PM10 + B30) increased the concentration of sweet potato root storage minerals: phosphorus by 152.5%, potassium by 77.4%, calcium by 205.5%, magnesium by 294.6%, iron by 268.4%, zinc by 228.6%, and sodium by 433.3%; compared to those in the control (PM10 + B30). This finding underscores the role of organic amendments in enhancing the nutritional quality of sweet potato roots^27^.

Moreover, the synergistic effects of combining poultry manure and biochar at various levels resulted in even greater enhancements in nutrient and mineral concentrations in sweet potato plants compared to their individual applications. This highlights the potential benefits of integrating multiple soil amendments to optimize nutrient management strategies in agricultural systems^28,29^. In addition, the interactive effects of factors such as year and site, year and amendments (poultry manure or biochar), and the combined application of poultry manure and biochar were found to notably affect the nutrient contents in sweet potato leaves and the mineral composition in the storage roots. These interactive effects of various factors on mineral concentrations in sweet potato storage roots further emphasize the complexity of nutrient dynamics in soil–plant systems. These interactions highlight the need for integrated management strategies that consider multiple factors to optimize nutrient uptake and crop productivity^30,31^. This suggests that synergistic interactions between environmental conditions and amendments can influence nutrient uptake by sweet potato plants. These findings are supported by studies highlighting the complex interactions between soil properties, amendments, and plant nutrition^32,33^.

The increase in concentrations of N, P, K, Ca, and Mg in sweet potato leaves, as well as mineral P, K, Ca, Mg, Fe, Zn and Na in sweet potato storage roots, due to the application of poultry manure and biochar, can be attributed to the increased availability of macronutrients. This increased availability is essential for soil fertility, as previously highlighted by Liu et al.^34^. The application of poultry manure and biochar improved soil nutrient availability, leading to greater nutrient uptake by sweet potato plants. The enhanced nutrient concentrations in the leaves and the increased mineral content in the storage roots of sweet potatoes, resulting from these applications, can be linked to improved soil quality, the release of nutrients into the soil solution, enhancements in chemical properties, the presence of beneficial organisms, and a more balanced nutritional status for the plants^4,21,24–26^.

The observed increase in nutrient concentrations in sweet potato leaves and the minerals stored in sweet potato storage roots can be attributed to the synergistic effects of poultry manure and biochar^4,29^. Poultry manure supplies essential macronutrients and micronutrients crucial for plant growth and development, as highlighted by Kingery et al.^12^. Biochar, on the other hand, enhances soil fertility and nutrient availability due to its porous structure, high cation exchange capacity, and ability to retain water and nutrients^30,32^. The combined application of poultry manure and biochar creates an optimal soil environment that promotes nutrient uptake and translocation within sweet potato plants, in line with the findings of Liu et al.^34^. Recent studies align with findings on the impact of poultry manure and biochar on sweet potato storage roots, supporting the significant increase in mineral concentrations observed. According to Alomari et al.^35^, the application of poultry manure and biochar, either separately or in combination, resulted in a substantial increase in phosphorus, potassium, calcium, magnesium, and sodium contents in lettuce crops. This outcome underscores the effectiveness of this combined amendment in alleviating nutrient deficiencies and enhancing plant nutrition in degraded soils, due to improved nutrient availability. However, a dissenting view was presented by Ayito et al. (2024)^36^, who argued that while poultry manure and biochar applications had positive effects on the mineral content, the efficacy varied based on the soil type and climatic conditions. This dissent emphasizes the need for a nuanced understanding of the contextual factors influencing the outcomes of such agricultural practices.

All treatments involving poultry manure and biochar, irrespective of the application rate, generated higher net profits compared to the control. The most notable increase in profitability was seen with the treatment PM10 + B30, which not only provided the highest sweet potato yield and quality but also demonstrated the most cost-effective approach for sweet potato production. The substantial net returns and high value-to-cost ratio underscore the financial benefits of using higher rates of poultry manure and biochar in sweet potato cultivation in severely degraded soils.

## Conclusion

The application of poultry manure, biochar, and their combination to degraded sand and sandy loam soils led to increased nutrient concentrations in sweet potato leaves and minerals stored in the storage roots. This enhancement is attributed to synergistic interactions between poultry manure and biochar. Among the various application rates tested, the most effective combination was found to be poultry manure at 10.0 t ha^−1^ and biochar at 30.0 t ha^−1^. This specific combination not only increased the nutritional quality of sweet potato but also maximized its economic returns. Hence, this application rate is recommended for soil fertility management, ensuring nutritional sustainability, and optimizing sweet potato performance in the forest savanna transition zone of southwest Nigeria. Enriching soil with poultry manure and biochar holds promise for enhancing nutrient concentrations in sweet potato leaves and mineral levels in storage roots, with potential implications for sustainable agriculture, soil restoration, and crop nutrition in degraded environments. However, further research is needed to validate these findings across diverse soil types, crops, agroecological zones, and land-use patterns due to spatial heterogeneity. Long-term field studies focusing on biochar's persistence in soils and its impact on crops are recommended. Additionally, exploring various application rates of poultry manure and biochar, as well as their combined use on soils and crops, is essential for future research.

## Materials and methods

### Study area and treatments

Field experiments were carried out in Owo, Ondo State, Nigeria, during the cropping seasons of 2019 and 2020 from April to August each year. These experiments were conducted at two sites, namely Rufus Giwa Polytechnic (site A) and Obasooto village (site B). Owo is situated in the transition zone between the forest and savanna, with geographic coordinates of approximately 7°12’N latitude and 5°35’E longitude, at an elevation of 348 m above sea level. The soil in Owo belongs to the Okemesi Series^37^ (Smyth and Montgomery, 1962) and is classified as an Alfisol (Oxic Tropuldalf)^38^ (Soil Survey Staff, 2014) or Luvisol^39^ (IUSS Working Group WRB, 2015). This soil type originates from quartzite, gneiss, and schist. The average annual rainfall in this region is approximately 1400 mm, while the mean annual temperature is approximately 32 °C. The sites had been left fallow for a year following rotational arable cropping, and no fertilizer, manure or biochar treatment had been applied to any of the sites in the previous six years. Historical soil management practices included conventional tillage methods such as ploughing, harrowing, and ridging.

Poultry manure (PM) at rates of 0, 5.0, and 10.0 t ha^−1^), along with varying levels of biochar (B) at 0, 10.0, 20.0, and 30.0 t ha^−1^, were combined in a 3 × 4 factorial layout. These twelve treatments were arranged in a randomized complete block design, with three replications. Each block consisted of 12 plots, each measuring 5 × 4 m. The blocks were spaced 1 m apart, and the plots were spaced 0.5 m apart. Crop establishment activities were carried out annually in April, and the same site was used consistently throughout the two-year study period.

### Land preparation, incorporation of poultry manure and biochar and planting of sweet potato vines

The experimental sites were prepared by slashing the vegetation with a cutlass followed by removing the weeds present. The trial plots were then laid out in accordance with the designated size of 5 × 4 m. The soils were then tilled to a depth of 20 cm with a handheld hoe. Poultry manure (PM) and biochar (B) were weighed and evenly spread over the soil at the specified rates (PM: 0, 5.0 and 10.0 t ha^−1^; B: 0, 10.0, 20.0 and 30.0 t ha^−1^). These soil amendments were incorporated into the soil to a depth of approximately 10 cm using a handheld hoe, two weeks prior to planting the sweet potato vines. This procedure was repeated in the second year of the experiment. Following soil preparation, sweet potato (*Ipomoea batatas* L. local variety) vines approximately 40 cm long, were planted in April of each experimental year. One sweet potato vine was planted per hole at a spacing of 1 m × 1 m, resulting in a sweet potato population of 20 plants per plot and 10,000 plants per hectare. The field plots were manually weeded twice, at 3 and 8 weeks after planting (WAP), to prevent weeds from competing with the crops for nutrients, water, and sunlight. During the trial, no irrigation water was applied. Chemical fertilizer or manure or biochar were also not applied during the field experiment.

### Soil analysis

Prior to the experiment in 2019, soil samples were taken from a depth of 0–15 cm at 10 randomly selected points across the experimental site. During the harvest in 2019 and 2020, additional disturbed soil samples were randomly collected from the center of each plot at five points per plot, also at a depth of 0–15 cm. All collected soil samples were bulked, air-dried, and sieved through a 2-mm sieve for routine chemical analysis, following the methods described by Carter and Gregorich (2007)^40^. Particle-size analysis was determined using the hydrometer method, and the textural class was determined using a textural triangle. Soil pH was measured in a soil/water (1:2) suspension with a digital electronic pH meter. The soil organic carbon content was determined using the Walkley–Black procedure involving dichromate wet oxidation. Total nitrogen (N) content was measured using micro-Kjeldahl digestion and distillation techniques. Available phosphorus (P) content was determined by Bray-1 extraction followed by molybdenum blue colorimetry. Exchangeable potassium (K), calcium (Ca), and magnesium (Mg) were extracted with a 1 M ammonium acetate (NH4OAc) solution at pH 7. Exchangeable K was analyzed using a flame photometer, while exchangeable Ca and Mg were measured with an atomic absorption spectrophotometer.

### Poultry manure and biochar preparation and analysis

The poultry manure (PM) used in this study was sourced from the poultry unit of the Teaching and Research Farm at Rufus Giwa Polytechnic, Owo, Ondo State. To facilitate mineralization, the poultry manure was subjected to a composting process lasting three weeks. The biochar materials employed in the experiments were derived from hardwood sources, including *Parkis biglosa*, *Khaya senegalensis*, *Prosopis africana*, and *Terminalia glaucescens*. These biochar materials were acquired from a local charcoal producer in Owo, Ondo State, Nigeria, who traditionally produces charcoal for domestic use. The carbonization process for the biochar was monitored using a thermocouple thermometer, and the kiln maintained an average temperature of 580 °C after 24 h. Both poultry manure and biochar were selected as soil amendments due to their wide availability and sustainability within the region.

Small subsamples of approximately 5 g each of the processed poultry manure and biochar materials used in the experiments were subjected to analysis to determine their nutrient compositions. Prior to analysis, both the poultry manure and biochar samples were prepared by air-drying, crushing, and sieving through a 2-mm sieve. These subsamples underwent various analyses, including measurements of electrical conductivity (EC), pH, ash content, organic carbon (OC), total nitrogen (N), phosphorus (P), potassium (K), calcium (Ca), and magnesium (Mg), as well as the concentrations of trace elements such as copper (Cu), iron (Fe), manganese (Mn), zinc (Zn), and sodium (Na).

The EC and pH of both the poultry manure and biochar were determined in a 1% (w/v) suspension in deionized water prepared by shaking at 100 rpm for 2 hours^41^. The ash content, expressed on a dry weight basis, was determined by combusting poultry manure and biochar samples in a muffle furnace at 750 °C for 6 h, following the ASTM D1762-84 (2021)^42^ standard. The OC content and total N, P, K, Ca, and Mg were determined using established procedures, such as the Walkley and Black method for OC content and micro-Kjeldahl digestion for total N. Other nutrients, including P, K, Ca, and Mg, were determined through wet digestion using a mixture of HNO_3_-H_2_SO_4_-HClO_4_ acids. Specifically, P was extracted using a Bray-1 solution and analyzed by molybdenum blue colorimetry, while exchangeable K, Ca, and Mg were extracted using a 1 M ammonium acetate, pH 7 solution. The exchangeable K was measured with a flame photometer, and Ca and Mg were determined using an atomic absorption spectrophotometer. To determine the concentrations of trace elements, such as Cu, Fe, Mn, Zn, and Na, samples of poultry manure and biochar with known quantities were incinerated at 760 °C in a muffle furnace. The resulting ash was treated with HCl, diluted with deionized water, and then analyzed for trace element concentrations. Cu, Fe, Mn, and Zn levels were determined in both poultry manure and biochar samples through the use of an atomic absorption spectrophotometer. The concentration of Na in these samples was measured using a flame photometer.

### Analysis of sweet potato leaves

During the cropping seasons of 2019 and 2020, sweet potato leaves were collected randomly from five plants in each plot for chemical analysis. These leaves, which were in the 2- to 3-week age range, were harvested 90 days after planting. The preparation of the samples involved a series of steps: the samples were initially dried in an oven set at 80 °C for 48 h and subsequently ground using a Willey-mill. To determine the levels of nitrogen (N) in the leaves, the micro-Kjeldahl digestion method was used. In addition to nitrogen analysis, the samples were subjected to a dry ashing process at 500 °C for 6 h in a furnace. A nitric-perchloric-sulphuric acid mixture was used to extract phosphorus (P), potassium (K), calcium (Ca), and magnesium (Mg). Phosphorus levels were determined through the colorimetric vanadomolybdate method, while potassium was quantified using a flame photometer. Calcium and magnesium levels were assessed using the ethylene diamine tetraacetic acid (EDTA) titration method^43^.

### Analysis of the mineral composition in sweet potato storage roots

In both years, an examination was conducted to analyze the mineral composition in the storage roots of sweet potato. After a growth period of 5 months, ten central plants from each plot were harvested at the two experimental sites. Subsequently, five storage roots of uniform size were chosen at random, washed, and then peeled. These storage roots were then collectively mixed and chopped into small pieces. The resulting chips were meticulously blended to create a consistent sample of root tissue derived from the five initial storage roots. A 100-g sample was selected and subjected to a 24-h drying process in an oven at 60 °C. Afterward, the dried samples were ground and securely stored in airtight containers for subsequent chemical analysis. To determine the levels of mineral elements, including phosphorus, potassium, calcium, magnesium, iron, and zinc, standard methods endorsed by the Association of Official Analytical Chemists^43^ were used. The samples were digested using a mixture of HNO_3_, H_2_SO_4_, and HClO_4_, and the phosphorus content was measured through the molybdenum blue colorimetric technique. Potassium, calcium, magnesium, iron, and zinc contents were assessed via atomic absorption spectrophotometry, following the protocols detailed in AOAC^43^.

### Statistical analysis

The experiments followed a randomized complete block design, employing factorial layouts to test the main impacts of year (Y), site (S), poultry manure (PM), and biochar (B), as well as the interactions of Y × S, Y × PM, Y × B, PM × B, PM × S, B × S, and Y × S × PM × B on the nutrients in sweet potato leaves and the minerals in the storage roots. The data analysis utilized a two-way analysis of variance (ANOVA) performed using the SAS (Statistical Analysis System)^44^ statistical package version 9.4 and Microsoft Office Excel 2013 software packages. The treatment means were separated using Fisher’s least significant difference (LSD) test at a significance level of *P* < 0.05.

### Cost-to-benefit analysis

A cost-to-benefit analysis was performed to determine the relative economic returns of the treatments using 2019 and 2020 annual market prices. The analysis involved calculating the total yield and cost–benefit ratios in US dollars (1$US = N360.00 Nigerian currency in the year 2019 and N370.00 in the year 2020), based on the harvest from the central bed (1 m^2^) within each plot. The costs for farm services were sourced from the Oja Oba market in Owo Local Government Area of Ondo State, Nigeria.

### Ethical approval

I confirm that all the research meets ethical guidelines and adheres to the legal requirements of the study country.

### Supplementary Information

Supplementary Figures.

## Data Availability

All datasets generated and/or analysed during the current study are included in this article.

## References

[1] Ande OT, Huising J, Ojo AO, Azeez J, Are KS, Olakojo SA, Fademi IO, Ojeniyi SO (2017). Status of integrated soil fertility management (ISFM) in southwestern Nigeria. Int. J. Sustain. Agric. Res..

[2] Motsa N, Modi AT, Mabhaudhi T (2015). Sweet potato (*Ipomoea batatas* L.) as a drought tolerant and food security crop. South Afr. J. Sci..

[3] Etana G (2021). Growth, yield and nutritional quality of sweet potato (*Ipomoea batatas* (L.) Lam) varieties as influenced by fertilizer types and rates. A review. Soil Sci. Plant Nutr..

[4] Adekiya AO, Agbede TM, Aboyeji CM, Dunsin O, Simeon VT (2019). Effects of biochar and poultry manure on soil characteristics and the yield of radish. Sci. Hortic..

[5] Mpanga IK, Adjei E, Dapaah HK, Santo KG (2021). Poultry manure induced garden eggs yield and soil fertility in tropical and semi-arid sandy-loam soils. Nitrogen.

[6] Lehmann J, Joseph S (2015). Biochar for Environmental Management: Science, Technology and Implementation.

[7] Głąb T, Palmowska J, Zaleski T, Gondek K (2016). Effect of biochar application on soil hydrological properties and physical quality of sandy soil. Geoderma.

[8] Mohanraj R, Sivasankar S (2014). Sweet potato (*Ipomoea batatas* L.)—A valuable medicinal food: A review. J. Med. Food.

[9] Agbede TM, Oyewumi A, Adekiya AO, Adebiyi OTV, Abisuwa TA, Ijigbade JO, Ogundipe CT, Oladele SO, Olaogun O, Eifediyi EK (2022). Assessing the synergistic impacts of poultry manure and biochar on nutrient-depleted sand and sandy loam soil properties and sweet potato growth and yield. Exp. Agric..

[10] Olajide K, Ndubuaku UM, Baiyeri PK (2023). Effect of poultry manure application rates on and yield of Saba (*Saba senegalensis*) in southeastern Nigeria. Sci. Res. Assays.

[11] Hoover NL, Law JY, Long LAM, Kanwar RS, Soupir ML (2019). Long-term impact of poultry manure on crop yield, soil and water quality, and crop revenue. J. Environ. Manag..

[12] Kingery WL, Wood CW, Mullins GL, Williams JC (1994). Impact of long-term application of broiler litter on environmentally related soil properties. J. Environ. Qual..

[13] Adeyemo AJ, Akingbola OO, Ojeniyi SO (2019). Effects of poultry manure on soil infiltration, organic matter contents and maize performance on two contrasting degraded alfisols in southwestern Nigeria. Int. J. Recycl. Org. Waste Agric..

[14] Jeffery S, Verheijen FGA, van der Velde M, Bastos AC (2015). A quantitative review of the effects of biochar application to soils on crop productivity using meta-analysis. Agric. Ecosys. Environ..

[15] Cayuela ML, Sánchez-Monedero MA, Roig A, Hanley K (2013). Biochar and denitrification in soils: when, how much and why does biochar reduce N_2_O emissions?. Sci. Rep..

[16] Spokas KA (2010). Review of the stability of biochar in soils: Predictability of O: C molar ratios. Carbon Manag..

[17] Lehmann J, Gaunt J, Rondon M (2006). Bio-char sequestration in terrestrial ecosystems—A review. Mitig. Adapt. Strateg. Glob. Change.

[18] Mao J, Johnson RL, Lehmann J, Olk DC, Neves EG, Thompson ML (2012). Abundant and stable char residues in soils: Implications for soil fertility and carbon sequestration. Environ. Sci. Technol..

[19] Tittonell P, Giller KE (2013). When yield gaps are poverty traps: The paradigm of ecological intensification in African smallholder agriculture. Field Crops Res..

[20] Salem HM, Schott LR, Piaskowski J, Chapagain A, Yost JL, Brooks E, Kahi K, Johnson-Maynard J (2024). Evaluating intra-field spatial variability for nutrient management zone delineation through geospatial techniques and multivariate analysis. Sustainability.

[21] Solaiman ZM, Shafi MI, Beamont E, Anawar HM (2020). Poultry litter biochar increases mycorrhizal colonisation, soil fertility and cucumber yield in a fertigation system on sandy soil. Agriculture.

[22] Olowoake AA, Abioye TA, Ojo A (2021). Influence of biochar enriched with poultry manure on nutrient uptake and soil nutrient changes in *Amaranthus caudatus*. Afr. J. Org. Agric. Ecol..

[23] Are KS, Adelana AO, Fademi IO, Aina OA (2017). Improving physical properties of degraded soil: Potential of poultry manure and biochar. Agric. Nat. Resour..

[24] Nwangwu BC, Anedo EO (2021). Influence of combined biochar and poultry manure on selected soil chemical properties and ginger yield in an Ultisol of Umudike, south-east Nigeria. Niger. Agric. J..

[25] Gunes A, Inal A, Taskin MB, Sahin O, Kaya EC, Atakol A (2014). Effect of phosphorus-enriched biochar and poultry manure on growth and mineral composition of lettuce (*Lactuca sativa* L. cv.) grown in alkaline soil. Soil Use Manag..

[26] Inal A, Gunes A, Sahin O, Taskin MB, Kaya EC (2015). Impacts of biochar and processed poultry manure, applied to a calcareous soil, on the growth of bean and maize. Soil Use Manag..

[27] Antonious GF (2024). Impact of biochar and organic fertilizers on sweet potato yield, quality, ascorbic acid, *ß*-carotene, sugars, and phenols contents. Int. J. Environ. Health Res..

[28] Agegnehu G, Nelson PN, Bird MI (2016). The effects of biochar, compost and their mixture and nitrogen fertilizer on yield and nitrogen use efficiency of barley grown on a Nitisol in the highlands of Ethiopia. Sci. Total Environ..

[29] Naeem MA, Khalid M, Aon M, Abbas G, Amjad M, Murtaza B, Ahmad N (2017). Combined application of biochar with compost and fertilizer improves soil properties and grain yield of maize. J. Plant Nutr..

[30] Lehmann J, Joseph S (2015). Biochar for Environmental Management: Science.

[31] Partey ST, Preziosi RF, Robson GD (2014). Short-term interactive effects of biochar, green manure, and inorganic fertilizer on soil properties and agronomic characteristics of maize. Agric. Res..

[32] Hossain MZ, Bahar MM, Sakar B, Donne SW, Ok YS, Palansooriya KN, Kirkham MB, Chowdhury S, Bolan N (2020). Biochar and its importance on nutrient dynamics in soil and plant. Biochar.

[33] Schulz H, Glaser B (2012). Effects of biochar compared to organic and inorganic fertilizers on soil quality and plant growth in a greenhouse experiment. J. Plant Nutr. Soil Sci..

[34] Liu L, Li J, Wu G, Shen H, Fu G, Wang Y (2021). Combined effects of biochar and chicken manure on maize (*Zea mays* L.) growth, lead uptake and soil enzyme activities under lead stress. Peer J..

[35] Alomari LM, Ahmad Al-Issa T, Abdul-LatifKiyyam M, Al Tawaha AR (2024). The impact of biochar and compost as soil amendments, combined with poultry manure, on the growth, yield, and chemical composition of lettuce (*Lactuca sativa*). J. Ecol. Eng..

[36] Ayito E, John K, Benjamin OI, John NM, Mngadi S, Heung B, Abbey L, Agyeman PC, Moodley R (2024). Synergistic effects of biochar and poultry manure on soil and cucumber (*Cucumis sativus*) performance. A case study from the southeastern Nigeria. Soil Sci. Annu..

[37] Smyth, A. J. & Montgomery, R. F. Soils and Land Use in Central Western Nigeria. Ibadan, Nigeria: Government Printer, 265 (1962).

[38] Soil Survey Staff. Keys to soil taxonomy. 12th ed. United States Department of Agriculture, Natural Resources Conservation Service, Washington, DC (2014).

[39] IUSS Working Group WRB. World Reference Base for Soil Resources 2014, Update 2015. International soil classification system for naming soils and creating legends for soil maps. World Soil Resources Reports No. 106. FAO, Rome, Italy (2015).

[40] Carter, M. R. & Gregorich, E. G. Soil Sampling and Methods of Analysis, second ed. *Canadian Society of Soil Science*, 1264, (CRC Press, Taylor & Francis Group, Boca Raton, Florida, 2007).

[41] Cantrell KB, Hunt PG, Uchimiya M, Novak JM, Ro SK (2012). Impact of pyrolysis temperature and manure source on physicochemical characteristics of biochar. Bioresour. Technol..

[42] ASTM D1762-84. Standard test method for chemical analysis of wood charcoal. American Society for Testing and Materials. Conshohocken (2021).

[43] Association of Official Analytical Chemists. Official methods of analysis of the association of official analytical chemists. In 21st edn, (ed. Latimer, G. W.) (AOAC International. Gaithersburg, 2019).

[44] SAS Institute Inc., SAS 9.4 Statements: Reference. Cary, NC: SAS Institute Inc (2013).

