# Supplementary material for: Impacts of poultry manure and biochar amendments on the nutrients in sweet potato leaves and the minerals in the storage roots
Source: Sci Rep. 2024 Jul 18;14:16598. doi: 10.1038/s41598-024-67486-9 (PMC11258218; doi:10.1038/s41598-024-67486-9)
Supplement: Supplementary file 1 — Supplementary Figures. [file 41598_2024_67486_MOESM1_ESM.docx]

**Supplementary Material Figure S1.** Impact of site, poultry manure (PM) and biochar (B), and their combination on soil pH (water), organic carbon (OC) and total nitrogen (TN).

**Supplementary Material Figure S2.** Impact of site, poultry manure (PM) and biochar (B), and their combination on soil available phosphorus (P), exchangeable potassium (K), exchangeable calcium (Ca) and exchangeable magnesium (Mg).

**Supplementary Material Figure S3.** Impact of site, poultry manure (PM) and biochar (B), and their combination on leaf nitrogen (N) and phosphorus (P) concentrations of sweet potato. Vertical bars show standard errors at P < 0.05 probability level.

**Supplementary Material Figure S4.** Impact of site, poultry manure (PM) and biochar (B), and their combination on leaf potassium (K), calcium (Ca) and magnesium (Mg) concentrations of sweet potato. Vertical bars show standard errors at P < 0.05 probability level.

**Supplementary Material Figure S5.** Impact of site, poultry manure (PM) and biochar (B), and their combination on mineral phosphorus (P) of sweet potato tuber. Vertical bars show standard errors at P < 0.05 probability level.

**Supplementary Material Figure S6.** Impact of site, poultry manure (PM) and biochar (B), and their combination on mineral potassium (K), calcium (Ca) and magnesium (Mg) of sweet potato tuber. Vertical bars show standard errors at P < 0.05 probability level.

**Supplementary Material Figure S7.** Impact of site, poultry manure (PM) and biochar (B), and their combination on mineral iron (Fe), zinc (Zn) and sodium (Na) of sweet potato tuber. Vertical bars show standard errors at P < 0.05 probability level.
